# Polymorphisms in hormone-sensitive lipase and leptin receptor genes and their association with growth traits in Barki lambs

**DOI:** 10.14202/vetworld.2021.515-522

**Published:** 2021-02-26

**Authors:** Adel H. M. Ibrahim

**Affiliations:** Department of Animal Breeding, Desert Research Center, Cairo, Egypt

**Keywords:** Barki lambs, growth traits, hormone-sensitive lipase, leptin receptor

## Abstract

**Background and Aim:**

Marker-assisted selection has many advantages over conventional selection in animal breeding. The candidate gene approach has been applied to identify genetic markers associated with economically important traits in livestock. This study was established to investigate variation in the hormone-sensitive lipase (*HSL*) and leptin receptor (*LEPR*) genes, and their association with growth traits in Barki lambs.

**Materials and Methods:**

Records for birth weight (BW), pre-weaning average daily gain (ADG1), weaning weight (WW), post-weaning average daily gain (ADG2), and marketing weight (MW) were obtained from 247 Barki lambs. Polymerase chain reaction–single-stranded conformational polymorphism analyses were used to detect variation in exon 9 of *HSL* and exon 19 of *LEPR*. General linear models were used to test for associations between the variation in ovine *HSL* and *LEPR*, and growth traits.

**Results:**

The SSCP banding patterns for *HSL* showed three variants (*H1*, *H2*, and *H3*), which contained two nucleotide-sequence differences (c.1865C>T and c.2038T>C). Two SSCP banding patterns (*L1* and *L2*) were observed for *LEPR* and these contained two nucleotide-sequence differences (c.2800G>A and c.2978C>G). The *HSL* genotype showed no effect on the studied traits. The *LEPR* genotype was proven to have significant effects (p<0.05) on ADG2 and MW. The presence of the *L1* variant was associated (p<0.01) with decreased ADG2 and MW.

**Conclusion:**

The finding of an association between *LEP*R gene variation and growth rate after weaning in Barki lambs warrants efforts to improve this trait.

## Introduction

In Egypt, there are major problems associated with supplying sufficient food to the population, especially meat products. This has directed attention toward improving the productivity of livestock adapted to the conditions of arid and semi-arid areas, which constitute about 94% of Egypt’s land. In these areas, Barki sheep have advantages over large ruminants, in that they can utilize a wider diversity of plants and have a higher reproductive rate, allowing populations to recover more quickly than large ruminants. Developing the productivity of sheep from meat is very important to overcome the shortfall of meat products. The growth traits of lambs are important factors influencing the meat productivity of sheep. Rapid growth during the early stage of lamb life could compensate for some of the rearing costs and result in a higher net profit for sheep producers. Understanding the genetic mechanisms that control growth traits is crucial in improving meat productivity in sheep [1]. The genetic factors that regulate appetite, lipolysis, and energy homeostasis are important factors in controlling growth and weight gain [2]. Mutations in these genetic factors could disrupt energy homeostasis. Two of the most important factors in regulating energy intake and consumption are hormone-sensitive lipase (*HSL*) and leptin receptor (*LEPR*), which are responsible for transferring information related to controlling food intake, metabolism, lipolysis, and energy expenditure [3,4].

*HSL* is an intracellular enzyme predominantly found in white and brown adipose tissues, along with skeletal muscles, and intestinal mucosa, steroidogenic tissues including adrenals, ovaries, and testis, and pancreatic β cells. It plays a role in many metabolic processes, energy homeostasis, and steroidogenesis through its unique ability to hydrolyze a wide variety of substrates, such as all forms of acylglycerols (triglycerol, diacylglycerol, and monoacylglycerol), cholesteryl esters, steroid esters, para-nitrophenyl esters, and retinyl esters [5]. *HSL* is encoded by the *HSL* gene, which is located on chromosome 14 and contains 10 exons separated by 9 introns (GenBank Gene ID: 100169699).

Leptin is an adipocyte-secreted hormone that regulates food intake, energy expenditure, and body weight in mammals. It acts through the *LEPR*, a member of the cytokine receptor superfamily, and has been found on the surface of cells in many organs and tissues, including the hypothalamus, liver, heart, kidneys, lungs, small intestines, pituitary cells, testes, ovaries, spleen, pancreas, adrenal glands, and adipose tissue [6]. Leptin and its receptor are known to play a role in regulating a multitude of physiological processes, including appetite, lipid metabolism, energy expenditure, growth, reproduction, and immune function [7]. *LEPR* is encoded by the *LEPR* gene, located on chromosome 1, and contains 20 exons and 19 introns. Numerous studies have focused on variation in the *LEPR* gene and its association with performance traits in livestock.

Given the crucial roles played by *HSL* and *LEPR* in glucose and fat metabolism as well as energy expenditure, variation within these genes might cause variation in metabolic function and lipase function and result in variation in growth traits in Barki lambs. Against this background, the main objective of this study was to determine the variation in a region covering a portion of exon 9 of the *HSL* gene and that in a region covering a portion of exon 19 of the *LEPR* gene, as detected using polymerase chain reaction–single-stranded conformational polymorphism (PCR-SSCP) analysis. Another objective is to reveal associations between these genetic variations and variation in growth traits in Egyptian Barki lambs.

## Materials and Methods

### Ethical approval

The study was carried out under permission and the guidelines of Desert Research Center and the Ministry of Agriculture and Land Reclamation, Egypt.

### Study period and location

A total of 247 Barki lambs, born in four successive years (2014-2017) and reared at Mariout Research Station (belonging to the Desert Research Center and located in the northwest of Egypt), were genotyped. The genotyping was conducted at Molecular Genetics Laboratory of Animal Breeding Department, Desert Research Center, in 2018-2019.

### Phenotypes and blood collection

At birth, the lambs were ear-tagged and weighed. Lambs suckled their ewes until weaning (80-95 days). Live weights at weaning weight (WW) and marketing (marketing weight [MW]; 8-9 months) were recorded. Pre- and post-weaning average daily gains (ADG1 and ADG2) were estimated from these weights.

Blood samples were collected from the jugular vein of the phenotyped lambs using vacuum tubes treated with 0.25% EDTA, and stored at −20°C on DNA extraction. DNA was extracted using a genomic DNA extraction kit (Qiagen, Hilden, Germany).

### Polymerase chain reaction–single-stranded conformational polymorphism

Regions of *HSL2* and *LEPR* were amplified using pairs of primers (Table-1). The PCR mixture for each gene contained 50 ng of genomic DNA, 0.25 mM of each primer, 160 mM dNTPs (GenElute; Merck KGaA, Darmstadt, Germany), 1.5 μL of 10× polymerase buffer (including 1.5 mM MgCl_2_), 0.6 U *Taq* DNA polymerase (GenElute; Merck KGaA, Darmstadt, Germany), and deionized water up to a final volume of 15 mL. Thermal cycling was conducted using a Bio-Rad C 1000 touch thermal cycler (Bio-Rad, Hercules, CA, USA), and the thermal cycle parameters for both genes were 95°C for 3 min, followed by 35 cycles of 96°C for 30 s, 60°C (for *HSL*) or 61°C (for *LEPR*) for 30 s, and 72°C for 30 s. This was followed by final elongation for 5 min at 72°C.

**Table-1:** Primer sequences.

Gene	Region	Amplicon Size (bp)	Primer sequence	Accession number
Hormone sensitive lipase	Exon 9	367	F: 5’ CCACTGGGAACCAACTCCC 3’	NM_001128154.1
			F: 5’ TGTGCACAGGTGGCAGG 3’	
Leptin receptor	Exon 19	360	F: 5’ GCACACAGAATCAGCGACCT 3’	NM_001009763.1
			F: 5’ GGTGGAGAATGGTTGCTCAAG 3’	

A total of 15 μL of each PCR amplicon was denatured at 105°C for 7 min, rapidly chilled on wet ice, and then loaded onto the electrophoresis unit [Protein II xi cells (Bio-Rad, USA)]. The amplicons from the *HSL* gene were screened using 14% acrylamide:bisacrylamide (37.5:1) gels at 250 V and 25°C for 16 h, and the amplicons from the *LEPR* gene were screened using 14% acrylamide:bisacrylamide gels at 280 V and 15°C for 18 h. The method of Byun *et al*. [8] was used to stain gels.

### Sequencing and analysis of variant polymorphisms

Two amplicons from lambs with homozygous SSCP patterns were purified using a PCR clean-up kit (GenElute, Merck KGaA, Darmstadt, Germany). The purified amplicons were delivered to the Macrogen sequencing company (Seoul, South Korea), to be sequenced in both directions using the BigDye terminator tool. DNA sequences, alignments, translations, analysis, and comparisons were achieved using DNAMAN and DNASTAR software.

### Statistical analysis

Using the general linear mixed model in SAS (version 9.1), two sets of analyses were run to test the effect of *HSL*/*LEPR* gene variation on the studied traits. In the first, genotype was modeled as a class variable. In the second, absence/presence of the detected variants was collapsed into two categories. Variation in the *HSL*/*LEPR* gene, year of lambing, parity of ewe, and gender of lamb were included as fixed effects. Sire was included as a random effect. Age at weaning was fitted as a covariate in the model testing the effect of variation in the *HSL*/*LEPR* gene on ADG1 and WW; additionally, age at marketing was fitted as a covariate in the model testing the effect of variation in the *HSL*/*LEPR* gene on ADG2 and MW. The significant differences were further explored using a Duncan test at p<0.05.

The generalized statistical model was as follows:

Y1_ijklmn_ = u +R_i_+G_j_+P_k_+B_l_+T_m_+e_ijklmn_

Y2_ijklmno_ = u+R_i_+G_j_+P_k_ + B_l_+T_m_+bAW_n_+e_ijklmno_

Y3_ijklmno_ = u+R_i_+G_j_+P_k_+B_l_+T_m_+bAM_n_+e_ijklmno_

where,

*Y1* = the evaluated BW;

*Y2* = the evaluated ADG1 or WW;

*Y3* = the evaluated ADG2 or MW;

*u* = the overall mean;

*R_i_* = the random effect of *i^th^* sire;

*G_j_*= the fixed effect of *j^th^*of gender of lamb, *j*=1, 2;

*P_k_* = the fixed effect of *k^th^* of parity of ewe, *k* =1, …5;

*B_l_* = the fixed effect of *l^th^* of year of lambing, *l*=1, …3;

*T_m_* = the fixed effect of *m^th^ HSL*/*LEPR* genotype (*m*=1, 2, 3 for *HSL* or *m*=1, 2 for *LEPR*) or the fixed effect of the absence/presence of each *HSL*/*LEPR* variant (*m*=0, 1);

*bAW_n_* = the partial regression coefficient of ADG1/WW on age at weaning as a covariate;

*bAM_n_* = the partial regression coefficient of ADG2/MW on age at marketing as a covariate; and

*e_ijklmn /_ e_ijklmno_* = Random error; assumed N.I.D. (0, σ^2^ e).

## Results

### Sequence variation

**Figure-1:**
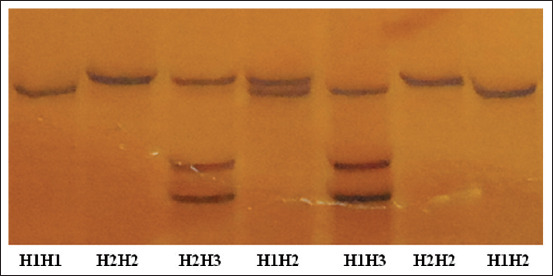
Polymerase chain reaction–single-stranded conformational polymorphism patterns for hormone-sensitive lipase gene.

**Figure-2:**
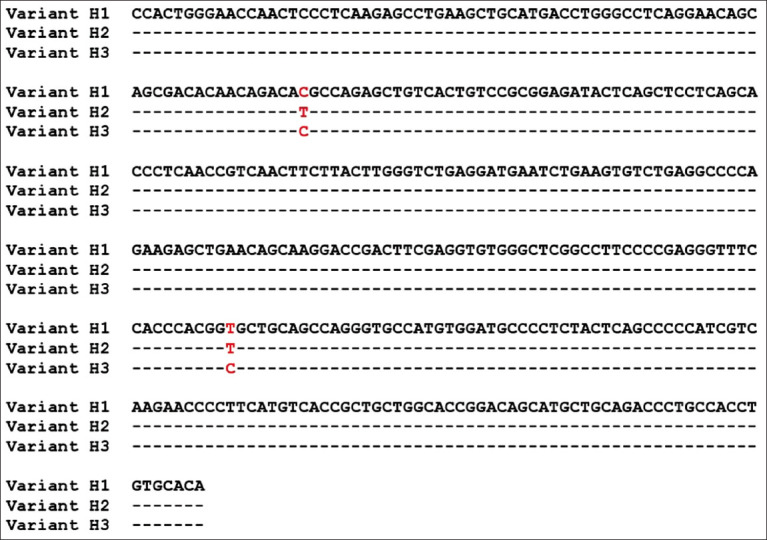
Sequences of the three variants of hormone-sensitive lipase (H1, H2, and H3).

Five genotypes were observed for *HSL* in the Barki lambs (Table-2 and Figure-1). These genotypes were derived from three sequence variants (Figure-2), which were named *H1*, *H2*, and *H3*. A total of two single-nucleotide polymorphisms (SNPs) (c.1865C>T and c.2038T>C) that were previously reported in the NBCI were detected by performing DNA sequencing of the three detected variants in exon 9 of the *HSL* gene. Notably, the c.1865C>T substitution results in the amino acid change threonine>methionine (p.Thr.622Met); however, the c.2038T>C substitution results in the amino acid change cysteine>arginine (p.Cys680Arg).

**Table-2:** Genotype frequencies in the amplified regions of *HSL and* *LEPR* in Barki lambs.

Gene	Parameter	Value			Parameter	Value				
*HSL*	Variant frequency (%)	H1 (65.4)	H2 (28.5)	H3 (6.1)	Genotype frequency (%)	H1H1 (48.6)	H1H2 (27.1)	H1H3 (12.1.)	H2H2 (6.5)	H2H3 (5.7)
*LEPR*	Variant frequency (%)	L1 (70.6)	L2 (29.4)		Genotype frequency (%)	L1L1 (54.3)	L1L2 (32.8)	L2L2 (12.9)		

HSL=Hormone-sensitive lipase, LEPR=Leptin receptor

**Figure-3:**
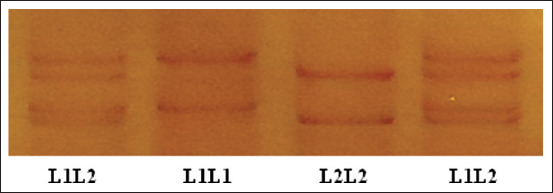
Polymerase chain reaction–single-stranded conformational polymorphism patterns for leptin receptor gene.

**Figure-4:**
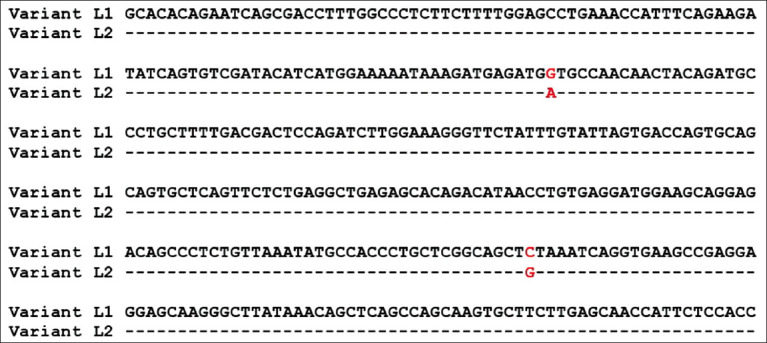
Sequences of the two variants of leptin receptor (L1 and L2).

As shown in Table-2 and Figure-3, two different SSCP banding patterns were observed for amplicons from the amplified region of *LEPR* in the Barki lambs and three combinations of SSCP patterns corresponding to three different genotypes were detected. These genotypes comprised two variant sequences (Figure-4), which were named *L1* and *L2*. The results of sequencing the two detected variants revealed two SNPs (c.2800G>A and c.2978C>G), which were previously reported in exon 19 of the *LEPR* gene. These are missense SNPs and do result in amino acid changes, namely, valine>methionine (p.Val934Met) and serine>cysteine (p.Ser993Cys), respectively.

### Effect of sire

The sire exhibited a significant effect on WW (p<0.005) and MW (p<0.01).

### Effect of gender

The gender of lamb showed a highly significant effect (p<0.001) on birth weight (BW), ADG1, WW, and MW. Male lambs were heavier than female lambs in BW, WW, and MW.

### Effect of non-genetic factors

Ewe parity significantly affected (p<0.001) BW, whereas the year of lambing did not affect any of the studied traits. Age at weaning showed a significant effect (p<0.01) on WW, whereas age at marketing had no significant effects on the studied traits.

### Effect of *HSL* gene variation

The results presented in Tables-3 and 4 show no significant association of ovine *HSL* exon 9 variants or genotypes with growth traits.

**Tables-3:** Least square means and their standard errors for growth traits in Barki lambs according to the hormone-sensitive lipase genotypes.

Trait	Genotype					Significance

	H1H1 (120)	H1H2 (67)	H1H3 (30)	H2H2 (16)	H2H3 (14)	
BW	3.48±0.05	3.50±0.07	3.63±0.11	3.75±0.12	3.27±0.12	0.130
ADG1	174.39±3.39	179.47±4.11	177.81±5.63	174.37±6.06	174.21±8.59	0.741
WW	19.65±0.34	20.12±0.44	20.16±0.66	19.77±0.64	19.79±0.83	0.779
ADG2	86.74±2.05	83.40±3.01	85.45±5.26	90.38±4.49	88.16±6.99	0.753
MW	43.31±0.70	42.86±0.98	43.41±1.48	44.30±1.53	44.06±2.36	0.955

BW=Birth weight, ADG1=Pre-weaning daily gain, WW=Weaning weight, ADG2=Post weaning daily gain, MW=Marketing weight

**Table-4:** Association of the absence/presence of hormone-sensitive lipase variants with growth traits in Barki lambs.

Trait	Variant being assessed	LSM±SE				Significance

		N	Absent variant	N	Present variant	
BW	H1	44	3.59±0.09	203	3.50±0.04	0.446
	H2	136	3.50±0.05	111	3.54±0.06	0.226
	H3	217	3.52±0.04	30	3.45±0.08	0.612
ADG1	H1	44	174.32±4.90	203	176.33±2.46	0.580
	H2	136	174.79±3.06	111	177.43±3.14	0.495
	H3	217	175.95±2.41	30	176.13±4.93	0.974
WW	H1	44	19.77±0.50	203	19.84±0.25	0.668
	H2	136	19.71±0.31	111	19.98±0.33	0.473
	H3	217	19.81±0.25	30	19.98±0.52	0.948
ADG2	H1	44	89.68±3.70	203	85.53±1.62	0.290
	H2	136	86.59±1.91	111	85.89±2.36	0.829
	H3	217	86.21±1.60	30	86.72±4.24	0.826
MW	H1	44	44.22±1.25	203	43.17±0.53	0.491
	H2	136	43.32±0.64	111	43.40±0.77	0.880
	H3	217	43.31±0.53	30	43.71±1.29	0.874

BW=Birth weight, ADG1=Pre-weaning daily gain, WW=Weaning weight, ADG2=Post weaning daily gain, MW=Marketing weight

### Effect of *LEPR* gene variation

The SSCP patterns at exon 19 of *LEPR* of 247 individuals were analyzed for correlations with growth traits (Table-5). Statistically significant results (p<0.05) were found for associations of ADG2 and MW values with *LEPR* genotypes; however, no significant associations of *LEPR* genotypes with BW, ADG1, and WW values were detected (p>0.05). Individuals with genotype *L2L2* had superior ADG2 and MW when compared with those with *L1L1* and *L1L2*. As shown in Table-6, the presence of the *L1* variant in the lamb genotype was associated (p<0.01) with decreases in ADG2 and MW.

**Table-5:** Least square means and their standard errors for growth traits in Barki lambs according to the leptin receptor genotypes.

Trait	Genotype			Significance

	L1L1 (134)	L1L2 (81)	L2L2 (32)	
BW	3.49±0.05	3.54±0.07	3.59±0.10	0.347
ADG1	174.64±3.11	176.69±3.85	179.75±5.04	0.754
WW	19.67±0.32	19.93±0.39	20.25±0.49	0.690
ADG2	84.14^b^±2.02	94.36^ab^±2.66	100.08^a^±2.95	0.012*
MW	42.61^a^±0.68	45.93^ab^±0.83	47.56^a^±1.11	0.017*

BW=Birth weight, ADG1=Pre-weaning daily gain, WW=Weaning weight, ADG2=Post-weaning daily gain, MW=Marketing weight.

* Refers to significance at (p<0.05)

**Table-6:** Association of the absence/presence of leptin receptor variants with growth traits in Barki lambs.

Trait	Variant being assessed	LSM±SE				Significance

		N	Absent variant	N	Present variant	
BW	L1	32	3.59±0.10	215	3.51±0.04	0.209
	L2	134	3.49±0.05	113	3.55±0.06	0.230
ADG1	L1	32	179.75±5.03	215	175.42±2.42	0.507
	L2	134	174.65±3.11	113	177.56±3.10	0.547
WW	L1	32	20.25±0.50	215	19.77±0.25	0.454
	L2	134	19.67±0.32	113	20.02±0.32	0.479
ADG2	L1	32	100.08±2.96	215	84.22±1.61	0.003**
	L2	134	84.14±2.03	113	88.81±2.18	0.156
MW	L1	32	47.56±1.11	215	42.73±0.53	0.005**
	L2	134	42.61±0.69	113	44.24±0.70	0.134

BW=Birth weight, ADG1=Pre-weaning daily gain, WW=Weaning weight, ADG2=Post-weaning daily gain, MW=Marketing weight.

** Refers to significance at (p<0.01)

The obtained results of the additive/dominance effects of *LEPR* variants on the growth traits are shown in Table-7. These results revealed a significant additive (p<0.01) effect for the *LEPR* genotype on ADG2 (5.878 g/day±2.076) and MW (1.889 kg±0.688).

**Table-7:** Genetic effects of the ovine leptin receptor gene on growth traits in Barki lambs.

Trait	Genetic effect			

	Additive	p value	Dominance	p value
BW	0.051±0.052	0.324	0.028±0.079	0.722
ADG1	2.415±3.110	0.438	1.063±4.697	0.821
WW	0.281±0.317	0.376	0.149±0.480	0.756
ADG2	5.878±2.076	0.005**	–2.854±3.177	0.370
MW	1.889±0.688	0.006**	–0.637±1.053	0.546

BW=Birth weight, ADG1=Pre-weaning daily gain, WW=Weaning weight, ADG2=Post-weaning daily gain, MW=Marketing weight.

** Refers to significance at (p<0.01)

## Discussion

### Effect of *HSL* genotype on growth traits

This is the first report regarding the effect of *HSL* gene variation in Barki sheep. The SNPs detected in this study were not the same SNPs as were detected in Suffolk sheep [9], which were three single-nucleotide substitutions in intron 5 and one non-synonymous substitution in exon 9 of *HSL*. None of those substitutions was associated with post-weaning growth, whereas the detected substitutions in intron 5 were associated with eye muscle depth, eye muscle width, and fat depth above the eye muscle.

Various SNPs have been reported in other livestock, but only a few of them have been further analyzed. In cattle, two SNPs (c.276C>T and c.51C>T) have been found to be associated with carcass and meat quality traits in Chinese Simmental-cross steers [10]. Furthermore, three SNPs (rs109759779, rs109598915, and rs41887406), selected on the basis of evolutionary conservation, were studied and the obtained results suggested a possible association of SNP1 with the levels of oleic acid and total monounsaturated fatty acids (SFA) (p<0.01), and SNP2 and SNP3 with the level of heneicosylic acid (p<0.01) [11].

In goats, two synonymous polymorphisms were identified at exons 2 (c.327C>A>T) and 3 (c.558C>T) of the *HSL* gene [12]. The same study revealed a missense polymorphism at exon 6 (c.1162G>T), which involves an alanine to serine substitution at position 388.

In pigs, the influence of *HSL* gene polymorphism c.442G>A on carcass traits was investigated [13], and two alleles A and G were detected with frequencies of 0.738 and 0.262, respectively. Moreover, three genotypes AA, AG, and GG were found with frequencies of 0.700, 0.075, and 0.225, respectively. In the observed population, allele A was associated with better animal muscularity, while allele G was associated with greater fat content. In addition, the *HSL* variation in two local Chinese pig breeds (Nuogu bei Luobo and Large White and Landrace crossbreed) was also studied. The results confirmed that the variation in this gene significantly affected muscularity, loin area, and weight of ham [14]. The results also revealed a mutation (c.873G/A) at exon 2 of *HSL* in Shanzhu × Duroc crossbreeds, which was associated (p<0.05) with increased rib eye area and decreased intramuscular fat and backfat thickness.

In general, variation in exon 9 of *HSL* is not associated with growth traits. This might be due to the variation in the studied region not directly affecting the weight gain of animals, instead altering the subcutaneous, and intramuscular fat composition of the body [8].

### Effect of *LEPR* genotype on growth traits

In this study, analysis of exon 19 in *LEPR* (Figure-4) revealed two missense mutations, the first of which (c.2800G>A) resulted in the amino acid substitution Val934Met, and the second of which (c.2978C>G) resulted in the amino acid substitution Ser993Cys. Tables-5 and 6 show significant associations of the variation in *LEPR* with growth speed after weaning (ADG2 and MW). There is a lack of reports on studies concerning the effect of the *LEPR* gene on growth traits in sheep in the literature; however, many previous reports have demonstrated associations between genetic variation in *LEPR* and other economically important traits. A previous study [15] sequenced a region covering exon 2 to exon 16 of the ovine *LEPR* gene using complementary DNA and identified two SNPs in exon 2 (T240C and T279C), one SNP (A16830G) in exon 10, and one SNP (C2373T) in exon 14. The SNP in exons 2 tended to associate with feed intake during gestation (p=0.087), whereas the SNP in exon 2 significantly (p=0.0229) associated with residual feed intake during lactation. Another study [16] detected three different mutations in the coding region of the *LEPR* gene: SNP A (chr1: 40787726C>T), which resulted in the amino acid change Arg62Cys; SNP B (chr1: 40857869C>T), which resulted in the amino acid change Pro1019Ser; and SNP C (chr1: 40858019A>G), and which resulted in the amino acid change Lys1069Gln, in Davisdal ewes. In Indonesian breeds of sheep (fat-tailed sheep, thin-tailed sheep, and Garut composite sheep), a novel SNP in the genomic region (g.40854778A>C) of the *LEPR* gene was also identified and found to be significantly (p<0.05) associated with SFA (including tricosanoic acid [C23:0] and tetracosanoic acid [C24:0]) and polyunsaturated fatty acid (docosahexaenoic acid (C22:6n3) [17].

Moreover, in cattle, variation in the *LEPR* gene was confirmed to have associations with body weight and ADG at 6 and 12 months of age in Nanyang calves [18]. In humans, several studies found associations of the polymorphisms of Lys109Arg in exon 4, Gln223Arg in exon 6, and Lys656Asn in exon 14 of the *LEPR* gene with body weight and body gain. The *LEPR* Arg109Lys and Arg223Gln polymorphisms were shown to be positively associated with weight gain in Dutch adults [19]. The weight-gainers with the Arg109 or Arg223 allele had higher leptin levels than those not carrying these alleles.

The effect of the *LEPR* genotype on growth traits after weaning might be due to the critical roles played by *LEPR* in the control of glucose metabolism and energy balance [20]. Energy balance has associations with whole-body mass and skeletal muscle mass through affecting the metabolism of whole-body protein as well as skeletal muscle protein. A negative energy balance causes decreases in skeletal muscle mass as a result of imbalanced rates of muscle protein synthesis and degradation. The previous work showed that the effect of energy deficits resulted in a 5-10% loss in initial body mass and a 19% decrease in the synthesis of skeletal muscle protein [21].

*LEPRs* are present on β cells as well as on muscle and fat cells, enabling leptin to modulate the secretion and action of insulin [22]. Insulin causes weight gain when the cells absorb too much glucose and the body converts this into fat [23]. Despite sheep being more tolerant of insulin than non-ruminants, insulin treatments have been reported to increase weight gain and fat deposition in sheep [24].

The effect of *LEPR* variation on growth traits after weaning might also be explained by other phenomena. Markedly, the *L2* variant, which is associated with higher ADG2 and heavier MW, carries the nucleotide substitutions c.2800G>A and c.2978C>G, which would lead to putative amino acid substitutions of valine to methionine and serine to cystine, respectively. Furthermore, the *L2* variant has a higher degree of additive effects because the *L2L2* homozygote was found to have a higher value of additive effect than the heterozygote *L1L2*, which, in turn, had a higher value than the *L1L1* homozygote. These findings might be due to the amino acid substitutions. The amino acid point mutations might change the structure of *LEPR* and thus its function, such as the affinity of binding to *LEPR*, the expression level of the protein, or the protein’s durability [25]. These effects could lead to variation in growth traits after weaning. Against this background, further studies are needed to verify the binding affinity of the mutants to *LEPR* as well as to determine the expression level of the *LEPR*.

## Conclusion

This study revealed associations of variation in the *LEPR* gene with ADG2 and MW. The detected variant might be used in breeding programs to produce animals with superior growth traits after weaning in Barki sheep in Egypt and elsewhere.

## Authors’ Contributions

AHMI conceived and designed the research, conducted the sample collection, processed samples in the Molecular Laboratory, carried out the data analysis and writing of the manuscript, edited the manuscript and approved the submitted version of the manuscript.

## References

[1] Gebreselassie G, Berihulay H, Jiang L, Ma Y (2020). Review on Genomic Regions and Candidate Genes Associated with Economically Important Production and Reproduction Traits in Sheep (Ovies aries). Animals (Basel).

[2] Chabowska-Kita A, Kozak L.P (2016). The critical period for brown adipocyte development:Genetic and environmental influences. Obesity (Silver Spring).

[3] Gale S, Castracane V, Mantzoros C (2004). Energy homeostasis, obesity and eating disorders:Recent advances in endocrinology. J. Nutr.

[4] Fan S, Say Y (2014). Leptin and leptin receptor gene polymorphisms and their association with plasma leptin levels and obesity in a multi-ethnic Malaysian suburban population. J. Physiol. Anthropol.

[5] Iglesias J, Lamontagne J, Erb H, Gezzar S, Zhao S, Joly E, Truong V.L, Skorey K, Crane S, Madiraju S.R, Prentki M (2016). Simplified assays of lipolysis enzymes for drug discovery and specificity assessment of known inhibitors. J. Lipid Res.

[6] Wasim M, Awan F.R, Najam S.S, Khan A.R, Khan H.N (2016). Role of leptin deficiency, inefficiency, and leptin receptors in obesity. Biochem. Genet.

[7] Ramos-Lobo A.M, Donato J (2017). The role of leptin in health and disease. Temperature.

[8] Byun S, Fang Q, Zhou H, Hickford J (2009). An effective method for silver-staining DNA in large numbers of polyacrylamide gels. Anal. Biochem.

[9] Yang G, Forrest R, Zhou H, Hickford J (2014). Variation in the ovine hormone-sensitive lipase gene (HSL) and its association with growth and carcass traits in New Zealand Suffolk sheep. Mol. Biol. Rep.

[10] Fang X, Zhang L, Yu X, Li J, Lu C.Y, Zhao Z, Yang R (2013). Association of HSL gene E1-c. 276C>T and E8-c.51C>T mutation with economical traits of Chinese Simmental cattle. Mol. Boil. Rep.

[11] Goszczynski D, Mazzucco J, Ripoli M, Villarreal E, Rogberg-Muñoz A, Mezzadra C, Melucci L, Giovambattista G (2014). Characterization of the bovine gene LIPE and possible influence on fatty acid composition of meat. Meta Gene.

[12] Zidi A, Fernandez-Cabanas V.M, Carrizosa J, Jordana J, Urrutia B, Polvillo O, Gonzalez-Redondo P, Gallardo D, Amills M, Serradilla J.M (2010). Genetic variation at the goat hormone-sensitive lipase (LIPE) gene and its association with milk yield and composition. J. Dairy Res.

[13] Peciulaitienė N, Miceikienė I, Makstutienė N, Miseikienė R, Morkuniene K, Indriulytė-Bizienė R, Zalionytė E (2018). Lipe gene polymorphism c. 442 G>A influence on carcass traits in pigs. Biotech. Anim. Husbandry.

[14] Wang W, Xue W, Zhou X, Zhang L, Wu J, Qu L, Jin B, Zhang X, Ma F, Xu X (2012). Effects of candidate genes'polymorphisms on meat quality traits in pigs. Acta Agric. Scand. A.

[15] Jonas E, Martin G.B, Celi P, Li L, Soattin M, Thomson P.C, Raadsma H.W (2016). Association of polymorphisms in leptin and leptin receptor genes with circulating leptin concentrations, production and efficiency traits in sheep. Small Rumin. Res.

[16] Haldar A, French M.C, Brauning R, Edwards S.J, O'Connell A.R, Farquhar P.A, Davis G.H, Johnstone P.D, Juengel J.L (2014). Single-nucleotide polymorphisms in the LEPR gene are associated with divergent phenotypes for age at onset of puberty in Davisdale ewes. Biol. Reprod.

[17] Gunawan A, Pramukti F.W, Listyarini K, Abuzahra M.A, Karia J, Sumantri C, Inounu I, Uddin M.J (2019). Novel variant in the leptin receptor (LEPR) gene and its association with fat quality, odour and flavour in sheep. J. Indonesian Trop. Anim. Agric.

[18] Guo Y, Chen H, Lan X, Zhang B, Pan C, Zhang L, Zhang C, Zhao M (2008). Novel SNPs of the bovine LEPR gene and their association with growth traits. Biochem. Genet.

[19] van Rossum C.T, Hoebee B, van Baak M.A, Mars M, Saris W.H, Seidell J.C (2003). Genetic variation in the leptin receptor gene, leptin, and weight gain in young Dutch adults. Obes. Res.

[20] Yang Y, Niu T (2018). A meta-analysis of associations of LEPR Q223R and K109R polymorphisms with Type 2 diabetes risk. PLoS One.

[21] Pasiakos S.M, Vislocky L.M, Carbone J.W, Altieri N, Konopelski K, Freake H.C, Anderson J.M, Ferrando A.A, Wolfe R.R, Rodriguez N.R (2010). Acute energy deprivation affects skeletal muscle protein synthesis and associated intracellular signaling proteins in physically active adults. J. Nutr.

[22] D'souza A.M, Kieffer T.J (2017). Restoration of lepr in b cells of lepr null mice does not prevent hyperinsulinemia and hyperglycemia. Mol. Metab.

[23] Russell-Jones D, Khan R (2007). Insulin-associated weight gain in diabetes-causes, effects and coping strategies. Diabetes Obes. Metab.

[24] Cunningham H (1968). Effect of insulin and tolbutamide on growth rate, blood glucose and body composition of lambs. Can. J. Comp. Med.

[25] Ibrahim A.H.M (2019). Association of growth performance and body conformational traits with BMP4 gene variation in Barki lambs. Growth Factors.

